# Maximizing Nash Social Welfare Based on Greedy Algorithm and Estimation of Distribution Algorithm

**DOI:** 10.3390/biomimetics9110652

**Published:** 2024-10-24

**Authors:** Weizhi Liao, Youzhen Jin, Zijia Wang, Xue Wang, Xiaoyun Xia

**Affiliations:** 1 School of Information Science and Engineering, Jiaxing University, Jiaxing 314001, China; liaowz@zjxu.edu.cn (W.L.); xiaxiaoyun@zjxu.edu.cn (X.X.); 2 School of Computer Science and Cyber Engineering, Guangzhou University, Guangzhou 510006, China; 3 School Artificial Intelligence, Zhejiang Sci-Tech University, Hangzhou 310018, China; wangxuezstu@163.com

**Keywords:** Nash social welfare, greedy algorithm, estimation of distribution algorithm, neighborhood search

## Abstract

The Nash social welfare (NSW) problem is relevant not only to the economic domain but also extends its applicability to the field of computer science. However, maximizing Nash social welfare is an APX-hard problem. In this study, we propose two approaches to enhance the maximization of Nash social welfare. First, a general greedy algorithm (GA) capable of addressing the Nash social welfare problem for both agents with identical and differing valuations was presented. It is proven that the proposed algorithm aligns with the previous greedy algorithm when all agents possess identical valuations. Second, an innovative method for solving the Nash social welfare problems using evolutionary algorithms was developed. This approach integrates the Estimation of Distribution Algorithms (EDAs) with neighborhood search techniques to improve the maximization process of Nash social welfare. Finally, the proposed algorithms were implemented across a range of instances with the objective of maximizing Nash social welfare. The experimental results indicate that the approximation solutions derived from the Estimation of Distribution Algorithm outperform those obtained via the greedy algorithm.

## 1. Introduction

The fairness of the allocation of goods to agents is quantified by its Nash social welfare (NSW). Proposed by Nash for the bargaining problem, NSW serves as a unique objective that satisfies a set of natural axioms [1]. In addition to the economic domain, the equitable allocation of computing resources among users [2] and the scheduling of jobs in heterogeneous parallel computing environments to minimize execution time variance are also recognized as the Nash social welfare problem within the field of computer science. However, it is well known that maximizing the Nash social welfare is APX-hard. In this paper, we investigate the challenge of allocating a set of indivisible goods to agents with additive valuations in order to maximize Nash social welfare when agents possess either differing or identical valuations for the same goods. We present two algorithms aimed at maximizing Nash social welfare.

First, we introduce a general greedy algorithm designed to compute Nash social welfare based on two priority principles: the agent with lower valuations is prioritized in obtaining goods. Furthermore, once an agent has been granted priority access to goods, those unallocated goods that hold maximum value for that agent are allocated accordingly. Unlike the previous greedy algorithm, which is limited to addressing the Nash social welfare problem for agents with identical valuations, the proposed method extends its applicability to situations involving agents with differing valuations.

Second, we investigate a novel approach to solving the Nash social welfare problem using evolutionary algorithms [3,4,5]. Our method integrates the Estimation of Distribution Algorithm (EDA) with neighborhood search techniques to maximize Nash social welfare. Firstly, we proposed an encoding scheme for the allocation of goods to agents. Secondly, we introduce an algorithm for generating the initial population that guarantees each agent receives at least one good. Thirdly, we define an assignment probability matrix that characterizes the likelihood of allocating goods to agents. This distribution probability is updated by selecting superior solutions, and new individuals are generated through sampling from the probability matrix. Furthermore, we demonstrate that improvements in Nash social welfare can be achieved by exchanging and relocating goods among agents. To enhance the local search capability of our algorithm, we presented four distinct neighborhood search strategies.

In order to evaluate the effectiveness of the proposed algorithm, we conducted three sets of experiments. In the first set, both the greedy algorithm (GA) and the Estimation of Distribution Algorithm (EDA) were employed to address ten NSW problems under conditions where agents possess identical valuations. The experimental results indicate that the EDA outperforms the GA, with the approximate solution derived from the EDA exceeding 0.943 times that of the actual optimal solution. The second set examines the performance of GAs and EDAs when agents have different valuations. The Estimation of Distribution Algorithm was executed ten times for each NSW instance. The results demonstrate a significant improvement in solutions with EDAs compared to GAs. Furthermore, the standard deviation of outcomes obtained through EDAs is notably small, suggesting that this proposed method is both stable and effective. The third set of experiments investigated how sensitive EDAs are to key parameters.

The remainder of this paper is organized as follows: Section 3 introduces the concepts and preliminary information necessary for understanding our work. In Section 4, we present a greedy algorithm designed to maximize Nash social welfare in scenarios where agents possess either differing valuations or identical valuations for the same goods. We demonstrate that the previous greedy algorithm represents a specific instance of our proposed method when agents have identical valuations. In Section 5, we introduce an innovative approach to maximizing Nash social welfare through an evolutionary algorithm. This method integrates an Estimation of Distribution Algorithm (EDA) with neighborhood search techniques to enhance Nash social welfare outcomes. A distribution probability matrix is defined to characterize the assignment probability of goods among agents. The assignment probabilities are updated by selecting superior solutions, from which new individuals are generated via sampling from the probability. Additionally, four neighborhood search strategies are proposed to augment the local search capabilities of the algorithm. In Section 6, both the proposed greedy algorithm and the EDA are applied to address multiple NWS instances effectively. Finally, Section 7 concludes this paper with remarks on future research directions.

## 2. Related Work

In this section, we review related work on the fair allocation of a set of indivisible goods among agents with additive valuations for these goods. J. Garg et al. proposed an algorithm to compute market equilibria that can be rounded to yield the first constant-factor approximation algorithm for maximizing the Nash social welfare when agents have budget-additive valuation functions [1]. S. Branze et al. demonstrated that classic mechanisms exist that achieve outcomes closely approximating optimal Nash social welfare across all instances while simultaneously ensuring individual fairness; the quality of such mechanisms is measured by their price of anarchy [2]. A. Inoue et al. introduced a polynomial-time algorithm that maximizes the Nash social welfare within an additive error $\varepsilon v_{\max}$, where ε is an arbitrary positive number and $v_{\max}$ represents the maximum utility of a good [6]. However, evaluating the additive error in the output necessitates a nontrivial amount of effort. W. Li et al. developed a constant-factor approximation algorithm for Nash social welfare under submodular valuations, which constitutes one of the largest natural classes allowing for constant-factor approximations even in terms of additive welfare [7]. Nonetheless, it should be noted that the approximation ratio remains relatively large. S. Barman et al. presented a polynomial-time 288-approximation algorithm aimed at maximizing Nash social welfare under binary XOS valuations. For fair division instances involving binary subadditive valuations and a fixed constant *ε* ∈ (0, 1], exponentially many value queries are required to find an allocation yielding at least $\frac{1\;}{n^{1-\varepsilon }}$ in terms of Nash social welfare, where *n* denotes the number of agents [8]. However, the development of constant-factor approximation algorithms for *p*-mean (*p* ≤ 1) under binary XOS valuations remains an open problem. S. Barman et al. proposed the first sublinear approximation algorithm aimed at maximizing Nash social welfare under XOS valuations, which are specified via demand and XOS oracles. With access to both XOS and demand oracles for the valuations of *n* agents, one can compute in polynomial time an *O*(*n*^53/54^) approximation for the Nash social welfare maximization problem [9]. H. Akrami et al. presented a polynomial-time algorithm that achieves a 1.0345 approximation for maximization of Nash social welfare in instances where all agents possess 2-value additive valuations [10]. However, this proposed algorithm is specifically applicable only to scenarios involving agents with 2-value additive valuations. Xiaowei Wu et al. investigated the fairness aspect of Nash social welfare within budget-feasible allocation problems. Their findings indicate that a budget-feasible allocation that maximizes NSW attains a ¼ approximation of EF1; furthermore, this approximation ratio improves when item costs are relatively small compared to each agent’s budget [11]. R. Cole et al. proposed a polynomial-time algorithm that guarantees a constant-factor approximation of the geometric mean of the agent’s valuations and proved that the developed algorithm achieves an approximation factor of no greater than 2.889 [12]. J. Garg et al. presented two approximation algorithms which were named SMatch and RepReMatch for asymmetric agents with additive and submodular valuations, respectively. These approaches achieve approximation factors *O*(*n*) and *O*(*n*·log*n*) for additive and submodular valuations. However, these existing methods designed for the symmetric NSW problem fail to extend even to the highly restricted case where agent weights are either 1 or 2. Notably, S. Match does not provide a better guarantee in this case [13]. I. Caragiannis et al. demonstrated that their method selects allocations that are envy-free up to one good compelling notion that becomes quite elusive when combined with economic efficiency considerations. Their results indicate that maximizing Nash welfare guarantees each of the *n* agents at least a fraction of $2/(1+\sqrt{4n-3})$ of their maximum share guarantee; furthermore, each agent receives at least a 0.618 fraction of their pairwise maximum share guarantee [14]. S. Barman et al. developed two greedy algorithms aimed at optimizing Nash social welfare in two specific cases. The findings reveal that the greedy algorithm for identical valuations offers a 1.061-approximation guarantee when agents possess identical valuations; additionally, an exact solution can be obtained in polynomial time using the greedy algorithm for binary valuations [15]. However, scenarios involving agents with differing valuations for the same goods remain unaddressed. P. Jain and R.Vaish provide a systematic study of the computational complexity of maximizing NSW for many-to-one matchings under two-sided preferences [16]. W. Suksompong and N. Teh examined the issue of fairly allocating indivisible goods to agents, where weights represent their entitlements, and they propose a specific version of maximum weighted Nash welfare that can be implemented in polynomial time [17]. Y. Kawase et al. explored the characteristics of fair allocations when agents have binary valuations and analyzed the computational complexity associated with finding a fair allocation of mixed goods based on the proximity theorem [18]. S. Barman et al. presented a polynomial-time approximation algorithm for maximizing Nash social welfare in coverage instances [19]. A. Psomas and P. Verma. investigated the relationship between fairness and efficiency under a relaxation of truthfulness known as non-obvious manipulability [20]. S. Dai et al. examined the relation between a maximum Nash Social Welfare allocation and two well-adopted fairness properties and presented an algorithm for computing a pairwise maximin share allocation for identical variants [21]. G. Jugal et al. presented the first constant-factor approximation algorithm for the symmetric case under Rado valuations [22]. J. Garg and A. Murhekar employed well-known fairness notions of envy-freeness up to one good (EF1) and equitability up to one good (EQ1) in conjunction with Pareto optimality to solve the problem of fair and efficient allocation of a set of indivisible goods to agents with additive valuations [23].

The paper most closely related to our work is that of reference [15], which proposes two greedy algorithms aimed at maximizing the Nash social welfare (NSW) in two specific cases: one where agents have identical valuations and another where agents possess binary valuations. The algorithm named ALG-IDENTICAL in reference [15] is employed to address the NSW problem for scenarios involving agents with identical valuations. It has been demonstrated that the outcomes produced by ALG-IDENTICAL are at least 0.943 times that of the actual optimal solution. However, it is important to note that ALG-IDENTICAL is not applicable for solving the Nash social welfare problem when agents have differing valuations.

The advantages of those methods discussed in the aforementioned literature stem from their mathematical proof that the ratio of the approximate solution to the actual optimal solution remains constant. However, these methods are often effective only for specific Nash social welfare problems. In contrast to the existing approaches, evolutionary algorithms exhibit characteristics of self-learning and self-adaptation, enabling them to effectively address complex issues that the existing methods struggle to solve [24,25,26]. As a type of evolutionary algorithm, the Estimation of Distribution Algorithm (EDA) employs stochastic optimization techniques that explore potential solution spaces by constructing and sampling explicit probabilistic models based on promising candidate solutions [27,28,29,30]. Given that EDA utilizes a macro-level evolution strategy grounded in search space exploration, it demonstrates enhanced global search capabilities and faster convergence rates.

## 3. Preliminaries

In this section, we presented the relevant concepts and symbols associated with the Nash social welfare problem, followed by a formulation of the mathematical model for this issue.

Let *N* = {0, 1, …, *n* − 1} represent a set of agents and *M* = {0, 1, …, *m* − 1} denote a set of indivisible goods, where *m* > *n*. Each agent *i* ∈ *N* has an associated value *v*(*i*,*j*) for each unit of goods *j* ∈ *M*. An allocation *A* is expressed by *A*├*A*_0_ ∪ *A*_1_ ∪ … ∪ *A_n_*_−1_, where *A*_0_, *A*_1_, …, *A_n_*_−1_ are the set of goods allocated to agents 0 through *n* − 1, respectively, as shown in Equation (1). It follows that *A*_0_ ∪ *A*_1_ ∪ … ∪ *A_n_*_−1_ = *M*. For any two distinct agents *i*, *k* ∈ {0, 1, …, *n* − 1}; if *i* ≠ *k*, then we have *A_i_* ∩ *A_k_* = Ø.

(1) $$A=\left\{\begin{array}{l} A_{0}=\left[j_{0}^{1},j_{0}^{2},\ldots ,j_{0}^{l_{1}}\right]\\ A_{1}=\left[j_{1}^{1},j_{1}^{2},\ldots ,j_{1}^{l_{2}}\right]\\ A_{n-1}=\left[j_{n-1}^{1},j_{n-1}^{2},\ldots ,j_{n-1}^{l_{n-1}}\right] \end{array}\right.$$

The valuations assigned to the agents are assumed to be additive. The valuation of an agent *i* for *A_i_* is denoted by *V_i_*, and it is defined as follows:

(2) $$V_{i}=\sum_{j\in A_{i}}v(i,j)$$

Specifically, if *A_i_* = Ø, then *V_i_* = 0. The measure used to evaluate the quality of an allocation is its Nash social welfare, which represents the optimal geometric mean of valuations. For a given allocation *A*, the Nash social welfare of *A* is denoted as *NSW*(*A*) and is defined in Equation (3).

(3) $${\mathrm{NSW}\left(A\right)=(\prod_{i\in N}V_{i})\;}^{\frac{1}{n}}$$

The objective of the Nash social welfare problem is to identify an allocation that maximizes the geometric mean of agent valuations. This problem can be formally represented by the following mathematical model:

(4) $$\max{\left(\prod_{i\in N}V_{i}\right)}^{\frac{1}{n}}$$

*s.t.* (1) *A*_0_, *A*_1_, …, *A*_*n*−1_ ⊆ *M*

(2) *A*_0_ ∪ *A*_1_ ∪ … ∪ *A*_*n*−1_ = *M*

(3) *A_i_* ∩ *A_k_* = Ø, where *i*, *k* ∈ *N* and *i* ≠ *k*

## 4. Greedy Algorithm for Maximizing Nash Social Welfare

Let A^*^ be the Nash optimal solution. An allocation *B* is define as a 1/*β* approximation (where 0 ≤ *β* ≤ 1) if *NSW*(*B*) ≥ *β*·*NSW*(A^*^) [15]. S. Barman et al. proposed a greedy algorithm named ALG-IDENTICAL, which aims to maximize Nash social welfare when agents have identical valuations. The ALG-IDENTICAL algorithm guarantees a 1.061 approximation. However, it is important to note that the ALG-IDENTICAL algorithm is not applicable for solving the Nash social welfare problem in scenarios where agents have differing valuations. In this section, we presented a general greedy algorithm designed to maximize Nash social welfare, applicable in scenarios where agents possess either identical or different valuations. The proposed greedy algorithm is named ALG-GENERAL.

The allocation process of ALG-GENERAL is shown in Algorithm 1, which can be divided into two main sub-processes: the order-first sub-process to rank the value of goods in descending order for each agent (line 1–3) and the allocate-second sub-process to allocate goods to each agent (line 4–9).

First, the value of all goods of each agent is sorted in descending order. Then, the second loop in Algorithm 1 (line 5–9) is employed to allocate goods to an agent. In each iteration, the algorithm first finds the agent *i* with the lowest *Vi* (line 6). Subsequently, the goods that hold the highest value for the agent among the unassigned goods are identified (line 7) and allocated accordingly (line 8).

It is evident that ALG-GENERAL operates within polynomial-time complexity.
**Algorithm 1:** Greedy Algorithm for Maximizing Nash Social Welfare (ALG-GENERAL)
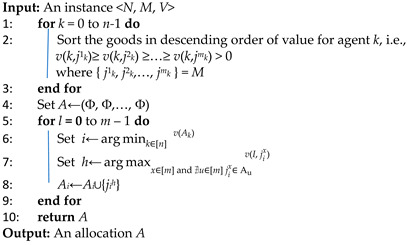


Next, an example is given to illustrate the operation of the proposed algorithm. Let *N* = {*X*, *Y*, *Z*} represent a set of agents, and let *M* = {*a*, *b*, *c*, *d*, *e*, *f*, *g*, *h*} denote a set of indivisible goods. The notation *X*-*M* = {*a*:3, *b*:8, *c*:11, *d*:10, *e*:1, *f*:5, *g*:4, *h*:6} represents the value of each good to agent *X*. Each element in the set *X*-*M* reflects the value of each good from the perspective of agent *X*. For instance, the notation *a*:3 signifies that the good *a* holds a value of 3 for agent *X*. Furthermore, the value of each good to agent *Y* and agent *Z* are as follows: *Y*-*M* = {*a*:2, *b*:10, *c*:11, *d*:9, *e*:3, *f*:6, *g*:5, *h*:8}; *Z*-*M* = {*a*:5, *b*:5, *c*:7, *d*:13, *e*:2, *f*:8, *g*:6, *h*:10}. According to the proposed algorithm, the solution process for the Nash social welfare problem is outlined as follows:

(1) First, the value of all goods of each agent is sorted in descending order. The results are as follows:
  *S*-*X*-*M* = *c*:11→*d*:10→*b*:8→*h*:6→*f*:5→*g*:4→*a*:3→*e*:1
  *S*-*Y*-*M* = *c*:11→*b*:10→*d*:9→*h*:8→*f*:6→*g*:5→*e*:3→*a*:2
  *S*-*Z*-*M* = *d*:13→*h*:10→*f*:8→*c*:7→*g*:6→*b*:5→*a*:5→*e*:2
(2) We use *X*-*A*, *Y*-*A*, and *Z*-*A* to store goods that agent *X*, *Y*, and *Z* have already acquired. Initially, these sets are empty sets. Thus, *V_X_* = *V_Y_* = *V_Z_* = 0. Consequently, this step can randomly select an agent and assign a good to it. It is assumed that the selected agent is *X*. Since all goods have not yet been allocated, the good *c* with the highest value to *X* is selected for *X*. Thus, we have *X*-*A* = {*c*}, *Y*-*A* = Ø, and *Z*-*A* = Ø.
(3) Since *Y*-*A* = Ø, and *Z*-*A* = Ø, we have *V_Y_* = *V_Z_* = 0. Consequently, we must allocate goods to either agent *Y* or agent *Z*. We may as well allocate goods to agent *Y* first in this step. Since the good *c* with the highest value to *Y* has already been assigned to *X*, we select *b* for *Y* among the unselected goods. Therefore, we arrive at the following allocations: *X*-*A* = {*c*}, *Y*-*A* = {*b*}, and *Z*-*A* = Ø.
(4) Since only *Z*-*A* is empty, we must allocate the good *d* with the highest value to agent *Z* in this step. Thus, we have *X*-*A* = {*c*}, *Y*-*A* = {*b*}, and *Z*-*A* = {*d*}.
(5) It is clear that *V_X_* = 11, *V_Y_* = 10, and *V_Z_* = 13. According to the proposed algorithm, agent *Y* acquires the good *h*, which holds the highest value for *Y* among the unallocated goods. It follows that *X*-*A* = {*c*}, *Y*-*A* = {*b*, *h*}, and *Z*-*A* = {*d*}.
(6) According to *X*-*A* = {*c*}, *Y*-*A* = {*b*, *h*}, and *Z*-*A* = {*d*}, it follows that *V_X_* = 11, *V_Y_* = 18, and *V_Z_* = 13. Thus, agent *X* obtains the good *f*, which holds the highest value for *X* from the unallocated goods. Therefore, we have *X*-*A* = {*c*, *f*}, *Y*-*A* = {*b*, *h*}, and *Z*-*A* = {*d*}.
(7) Since *V_X_* = 16, *V_Y_* = 18, and *V_Z_* = 13, agent *Z* acquires the good *g* which holds the highest value for *Z* from the unallocated goods. Thus, we obtain *X*-*A* = {*c*, *f*}, *Y*-*A* = {*b*, *h*}, and *Z*-*A* = {*d*, *g*}.
(8) According to *X*-*A* = {*c*, *f*}, *Y*-*A* = {*b*, *h*}, and *Z*-*A* = {*d*, *g*}, we have *V_X_* = 16, *V_Y_* = 18, and *V_Z_* = 19. Since the value of *V_X_* is minimal, agent *X* acquires the good *a*. It follows that X-*A* = {*c*, *f, a*}, Y-*A* = {*b*, *h*}, and Z-*A* = {*d*, *g*}.
(9) In the final step, agent *Y* acquires the good *e*, which is the only unallocated good. Consequently, we arrive at the final allocation results: *X*-*A* = {*c*, *f, a*}, *Y*-*A* = {*b*, *h, e*}, and *Z*-*A* = {*d, g*}. In this step, all goods have been successfully allocated. The total value of the goods acquired by agent *X* amounts to 19, while agent *Y*’s acquisitions hold a value of 21; similarly, agent *Z* also receives goods valued at 19. The NSW is equal to 19.64.

Finally, we demonstrate that the algorithm proposed in this paper is equivalent to the ALG-IDENTICAL algorithm when agents possess identical valuations.

**Theorem** **1.** *ALG-GENERAL is equivalent to the ALG-IDENTICAL algorithm when agents have identical valuations.*

**Proof of Theorem** **1.** The function of Step 1 in ALG-IDENTICAL is to sort the goods in descending order of value. Similarly, the function of the first loop in our algorithm (lines 1–3) also involve ordering the goods in descending order of value for each agent. It is evident that when agents have identical valuations, the function performed by the first loop in our algorithm is equivalent to those executed by Step 1 of ALG-IDENTICAL. Step 6 (line 6) of our algorithm serves a purpose analogous to that of Step 4 in ALG-IDENTICAL, which determines which agent has the lowest valuation. Therefore, we need to demonstrate that Steps 7 and 8 (lines 7–8) in our algorithm are equivalent to Step 5 in ALG-IDENTICAL. Once this equivalence is established, we can subsequently prove that the two algorithms are indeed equivalent.

It is evident that Step 5 in ALG-IDENTICAL involves allocating a good *j_l_* to the agent with the lowest valuations at iteration *l*, where the good *j_l_* represents the most valuable goods at this iteration. We can conclude that the goods *j^h^_i_* in our algorithm correspond to the good *j_l_* in ALG-IDENTICAL when agents possess identical valuations at iteration *l*. We will demonstrate this conclusion through induction as follows:

(1) When *l* = 1, it indicates that all goods are unallocated prior to the first iteration. Consequently, the function of Step 7 (line 7) in our algorithm is designed to allocate the most valuable goods *j_i_^h^* to the agent with the lowest valuation. Conversely, Step 5 in ALG-IDENTICAL aims to assign the most valuable good *j*_1_ to the agent with the lowest valuation during the initial iteration. Therefore, when agents possess identical valuations, both goods *j_i_^h^* and *j*_1_ refer to the same goods.
(2) We assume that the function of Step 7 (line 7) in our algorithm is to allocate the good *j_k_* to the agent with the lowest valuations at iteration *k* when agents have identical valuations. Here, the good *j_k_* represents the most valuable goods among the top *k* goods. Since these top *k* goods have already been allocated in the first *k* iterations, it follows that during iteration *k* + 1, when agents have identical valuations, the (*k* + 1)th most valuable good is defined as the goods with maximum value among those unallocated. Consequently, the function of Step 7 (line 7) in our algorithm serves to allocate goods *j_k_*_+1_ to the agent with minimal valuations at iteration *k* + 1. This function is equivalent to that described in Step 5 of ALG-IDENTICAL.

Therefore, we conclude that our algorithm is equivalent to the ALG-IDENTICAL algorithm when agents have identical valuations. It is important to note that ALG-IDENTICAL represents merely a special case of our proposed algorithm.? □

## 5. Estimation of Distribution Algorithm for Maximizing Nash Social Welfare

The Estimation of Distribution Algorithm (EDA) is a stochastic optimization technique grounded in statistical principles. By sampling the search space and employing statistical learning, EDA can effectively identify optimal search regions, subsequently generating new high-quality individuals. In this section, we propose a novel approach to address the Nash social welfare problem based on EDAs. An algorithm that integrates the Estimation of Distribution Algorithm (EDA) with neighborhood search techniques to maximize Nash social welfare was developed. The assignment of goods to agents is achieved by updating and sampling from a probabilistic model. Furthermore, we demonstrate that the condition for improving Nash social welfare can be enhanced through the exchange and relocation of goods. To bolster the local search capability of our algorithm, we present four distinct neighborhood search strategies.

### 5.1. Encoding for Individuals

Each individual corresponds to an allocation in our EDA algorithm. Let *N* = {0, 1, …, *n* − 1} represent a set of agents and *M* = {0, 1, …, *m* − 1} denote a set of indivisible goods, where *m* > *n*. The encoding of an individual is illustrated in Equation (1).

### 5.2. Population Initialization for EDA

In order to ensure that the initial population of EDA is diverse and that the Nash social welfare (NSW) of any allocation is not equal to zero, our algorithm divides the initialization process into two stages. Firstly, we select an agent from those who have never received goods and randomly assign one unallocated good to this agent. This operation is repeated until each agent has received one good. Since every agent possesses at least one good, it follows that the NSW of the allocation cannot be zero. Secondly, we randomly select an agent again and assign another unallocated item to them. This process continues until all items are allocated. It is evident that the randomness inherent in this allocation method promotes diversity within the population. We outline our population initialization algorithm in Algorithm 2, where *Psize* is the size of the population of the EDA.
**Algorithm 2:** Population initialization for EDA
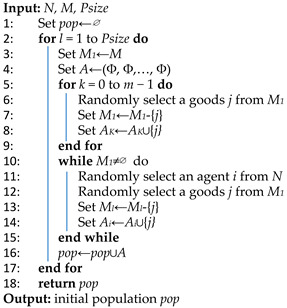


### 5.3. Probability Model and Its Updating and Sampling

Let *N* = {0, 1, …, *n* − 1} represent a set of agents and *M* = {0, 1, …, *m* − 1} denote a set of indivisible goods (*m* > *n*). We define the matrix *ρ*(*g*) presented in Equation (5) as the assignment probability model, where the dimensions of the matrix are *n* × *m*. The element *ρ_i_*_,*j*_(*g*) represents the probability of assigning good *j* to agent *i* at iteration *g*. Here, the variable *i* is an element of the set *N*, while *j* belongs to the set *M*.

(5) $$\rho \left(g\right)=\left[\begin{array}{ccc} \rho_{0,0}(g) & \cdots & \rho_{0,m-1}(g)\\ \vdots & \ddots & \vdots \\ \rho_{n-1,0}(g) & \cdots & \rho_{n-1,m-1}(g) \end{array}\right]$$

To guarantee a uniform sampling of the solution space during the initial phase of the proposed algorithm, we distribute the initial probability uniformly, as demonstrated in Equation (6).

(6) $$\rho \left(0\right)=\left[\begin{array}{ccc} \frac{1}{n} & \cdots & \frac{1}{n}\\ \vdots & \ddots & \vdots \\ \frac{1}{n} & \cdots & \frac{1}{n} \end{array}\right]$$

In order to enhance the suitability of the probability model for accurately representing the distribution of solution space and the evolutionary trends within the population, this algorithm selects the top *δ*% elite individuals that demonstrate the highest Nash social welfare from the overall population to update the probability. The probability matrix is updated in each iteration using Equation (7).

(7) $$\rho_{i,j}\left(g+1\right)=\left(1-\alpha \right)\rho_{i,j}\left(g\right)+\frac{\alpha }{\delta \%𢋅\mathrm{Psize}}\sum_{k=1}^{\delta \%\cdot \mathrm{Psize}}I_{i,j}^{k}(g)$$

Here, *α* ∈ (0,1) denotes a learning speed, and *Psize* represents the population size. The value of *I^k^_i_*_,*j*_(*g*) indicates whether a good *j* is assigned to an agent *i* in the *k*th elite individual. If a good *j* is assigned to an agent i, then we have *I^k^_i_*_,*j*_(*g*) = 1; otherwise, it holds that *I^k^_i_*_,*j*_(*g*) = 0.

(8) $$I_{i,j}^{k}\left(g\right)=\left\{\begin{array}{ll} 1 & a\;\mathrm{good}\;j\;\mathrm{is}\;\mathrm{assigned}\;\mathrm{to}\;\mathrm{an}\;\mathrm{agent}\;i\\ 0 & \mathrm{otherwise} \end{array}\right.$$

Here, *k* represents the *k*th individual in the elite population.

In the proposed algorithm, a new population is generated by sampling from the probability model at each iteration. The process of generating a new individual is as follows: An unallocated good is assigned to an agent through a roulette selection based on the assignment probability matrix. This operation is repeated until all goods are allocated, resulting in a new allocation being generated. Consequently, we can obtain new individuals through repeated sampling, thereby forming a new population for the next iteration.

### 5.4. Neighborhood Search Strategy

The global search capability of distribution estimation algorithms is robust; however, their local search capability tends to be relatively limited. The neighborhood search method is a systematic approach employed within a specific domain to identify the optimal solution through iterative stepwise refinement. To enhance the local search ability of the Estimation of Distribution Algorithm (EDA), we employ a neighborhood search method to improve an allocation. The Nash social welfare associated with an allocation can be improved through the exchange and movement of goods among agents. In this context, we propose four neighborhood search strategies aimed at strengthening the algorithm’s local search capacity by facilitating the exchange and relocation of goods between agents.

Let *A* = [*A*_0_, *A*_1_, …, *A_n_*_−1_] be an allocation. The Nash social welfare of *A* is defined as ${\left(\prod_{i\in N}V_{i}\right)}^{\frac{1}{n}}$. It is evident that a greater value of $\prod_{i\in N}V_{i}$ leads to a higher value of ${\left(\prod_{i\in N}V_{i}\right)}^{\frac{1}{n}}$. We present the following theorems.

**Theorem** **2.** Let A = [A_0_, A_1_, …, A_n−1_] denote an allocation. We define new allocations as follows: A^*^_i_ ←A_i_−{j} and A^*^_k_ *←A_k_* ∪{j}, where good j belongs to A_i_. Consequently, a new allocation denoted by A^*^ = [A^*^_0_, A^*^_1_, …, A^*^_n−1_] is obtained. If V_i_·v(k,j) − V_k_·v(i,j) − v(k,j)·v(i,j) > 0, then it follows that NSW(A^*^) > NSW(A).

**Proof of Theorem** **2.** It is straightforward to observe that if *m ≠ i*,*k*, then we have A^*^_m_ = *A_m_* for any *m* ∈ {0, 1, …, *n* − 1}. Therefore, we can express the following:

(9) $$\prod_{i\in N}V_{i}^{*}-\prod_{i\in N}V_{i}=\prod_{m\in N\wedge m\notin \left\{i,k\right\}}V_{m}\cdot (V_{i}^{*}{\cdot V}_{k}^{*}\;-\;V_{i}{\cdot V}_{k})$$

Since

(10) $$V_{i}^{*}=V_{i}-(i,j),\;V_{k}^{*}=V_{k}+v(k,j)$$

we obtain the following equation:

(11) $$\begin{array}{ll} V_{i}^{*}\cdot V_{k}^{*} & =(V_{i}-v(i,j))\times (V_{k}+v(k,j))\\ & =V_{i}\cdot V_{k}+V_{i}\cdot v(k,j)-v(i,j)\cdot V_{k}-v(i,j)\cdot v(k,j) \end{array}$$

This implies that

(12) $$V_{i}^{*}{\cdot V}_{k}^{*}-V_{i}\cdot V_{k}=V_{i}\cdot v(k,j)-v(i,j)\cdot V_{k}-v(i,j)\cdot v(k,j)$$

Thus, if $V_{i}$·*v*(*k*,*j*) − *v*(*i*,*j*)·$V_{k}$ − *v*(*i*,*j*)·*v*(*k*,*j*) > 0, we conclude that

(13) $$\prod_{i\in N}V_{i}^{*}-\prod_{i\in N}V_{i}\;>\;0$$

Hence, it follows that *NSW*(A^*^) > *NSW*(*A*). □

**Theorem** **3.** Let A = [A_0_, A_1_, …, A_n−1_] represent an allocation. We exchange good j_1_ from agent A_i_ with good j_2_ from agent A_k_. Specifically, we update the allocations as follows: A^*^_i_ *←(A_i_ − {j_1_})* ∪{j_2_}; A^*^_k_ *←(A_k_−{j_2_})* ∪{j_1_}. This results in a new allocation represented by A^*^ = [A^*^_0_, A^*^_1_, …, A^*^_n−1_]. Let x = V_i_, y = V_k_, a_1_ = v(i,j_1_), a_2_ = v(i,j_2_), b_1_ = v(k,j_2_), and b_2_ = v(k,j_1_). If x·(b_2_ − b_1_) − a_1_·(y − b_1_ + b_2_) + a_2_·(y − b_1_+ b_2_) > 0, we have NSW(A^*^) > NSW(A).

**Proof of Theorem** **3.** Since allocation A^*^ is derived by exchanging good *j*_1_ from agent *A_i_* with good *j*_2_ from agent *A_k_*, while the goods of other agents remain unchanged, we can express this relationship with the following equations:

(14) $$V_{i}^{*}=V_{i}-v(i,j_{1})+v(i,j_{2})$$

(15) $$V_{k}^{*}=V_{k}-v(k,j_{2})+v(k,j_{1})$$

Consequently, we obtain

(16) $$V_{i}^{*}{\cdot V}_{k}^{*}=x\cdot y+x\cdot (b_{2}-b_{1})-a_{1}\cdot (y-b_{1}+b_{2})+a_{2}\cdot (y-b_{1}+b_{2})$$

Given that *V_i_* ·*V_k_*= *x*·*y*, we can derive the following:

(17) $$V_{i}^{*}\cdot V_{k}^{*}-V_{i}\cdot V_{k}=x\cdot (b_{2}-b_{1})-a_{1}\cdot (y-b_{1}+b_{2})+a_{2}\cdot (y-b_{1}+b_{2})$$

Thus, if *x*·(*b*_2_ − *b*_1_) − *a*_1_·(*y* − *b*_1_ + *b*_2_) + *a*_2_·(*y* − *b*_1_ + *b*_2_) > 0, it follows that

(18) $$\prod_{i\in N}V_{i}^{*}-\prod_{i\in N}V_{i}\;>\;0$$

This indicates that *NSW*(A^*^) > *NSW*(*A*). □

It is important to note that neighborhood search does not guarantee that each operation will result in an increased Nash social welfare value. If the neighborhood search fails to identify a better value, the Nash social welfare value will remain unchanged. Therefore, the value of NSW can only be enhanced by moving a good when *V_i_*·*v*(*k*,*j*) − *V_k_*·*v*(*i*,*j*) − *v*(*k*,*j*)·*v*(*i*,*j*) > 0. Additionally, the value of NSW can only be increased through an exchange of goods when *x*·(*b*_2_ − *b*_1_)− *a*_1_·(*y* − *b*_1_ + *b*_2_)+ *a*_2_·(*y* − *b*_1_ + *b*_2_) > 0.

Since the neighborhood search method represents an advanced optimization of the current solution, its incorporation into EDAs will not compromise the outcomes of EDAs. However, incorporating neighborhood search methods into EDAs will lead to an increase in the runtime of EDAs. Consequently, it is essential to define the number of operations allocated for neighborhood search.

According to Theorem 2 and Theorem 3, it is feasible to achieve a higher Nash social welfare in allocation by facilitating the exchange or movement of goods among agents for a given allocation. Consequently, we propose four neighborhood search strategies based on the principles of good exchange and movement, as outlined below.

**Figure 1:**
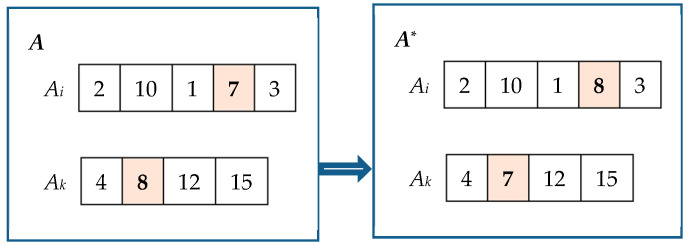
Random exchange good strategy.

**Figure 2:**
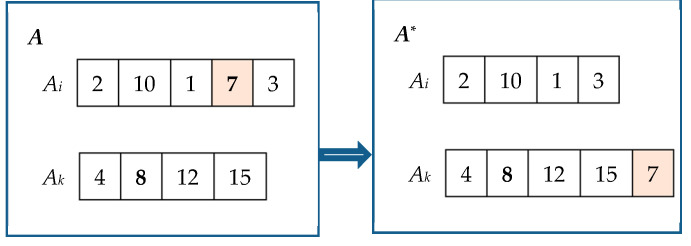
Random moving good strategy.

1. Random exchange good strategy. Let *A* = [*A*_0_, *A*_1_, …, *A_n_*_−1_] represent an allocation. Firstly, we randomly select *A_i_* and *A_k_* from the set *A*. Next, we randomly choose a good *j*_1_ from *A_i_* and a good *j*_2_ from *A_k_*. Subsequently, we exchange good *j*_1_ with *j*_2_. This process yields a new allocation denoted as A^*^. If *NSW*(A^*^) > *NSW*(*A*), allocation *A* is replaced by the new allocation. An illustrative example of this random exchange of goods is presented in Figure 1.
2. Random moving good strategy. Let *A* = [*A*_0_, *A*_1_, …, *A_n_*_−1_] represent an allocation. First, we randomly select *A_i_* and *A_k_* from *A*. Secondly, if *v*(*A_i_*) > *v*(*A_k_*) (*v*(*A_k_*) > *v*(*A_i_*)), we identify a good *j* from *A_i_* (*A_k_*), such that the following conditions hold: *V_i_*·*v*(*k*,*j*) − *V_k_*·*v*(*i*,*j*) − *v*(*k*,*j*)·*v*(*i*,*j*) > 0 (*V_k_*·*v*(*i*,*j*) − *V_i_*·*v*(*k*,*j*) − *v*(*i*,*j*)·*v*(*k*,*j*) > 0). Finally, we remove good *j* from *A_i_* (*A_k_*) and insert it into *A_k_* (*A_i_*). Consequently, we obtain a new allocation denoted as A^*^. We then replace the original allocation with this new one. An example of the random moving good strategy is illustrated in Figure 2.
3. The strategy of exchanging goods between maximum agent and minimum agent. Let *A* = [*A*_0_, *A*_1_, …, *A_n_*_−1_] denote an allocation. Define $i=\arg\max_{h\in [n]}^{v(A_{h})}$ and $k=\arg\min_{h\in [n]}^{v(A_{h})}$. We randomly choose a good *j*_1_ from *A_i_* and a good *j*_2_ from *A_k_* and exchange *j*_1_ with *j*_2_ to obtain a new allocation denoted by A^*^. If *NSW*(A^*^) > *NSW*(*A*), we use allocation A^*^ to replace allocation *A*.
4. The strategy of moving goods from maximum agent to minimum agent. Let *A* = [*A*_0_, *A*_1_, …, *A_n_*_−1_] represent an allocation. Define $i=\arg\max_{h\in [n]}^{v(A_{h})}$ and $k=\arg\min_{h\in [n]}^{v(A_{h})}$. We find a good *j* from *A_i_* such that *V_i_*·*v*(*k*,*j*) − *V_k_*·*v*(*i*,*j*) − *v*(*k*,*j*)·*v*(*i*,*j*) > 0. Subsequently, we remove good *j* from *A_i_* and insert it into *A_k_*. This process generates a new allocation denoted as A^*^, which replaces the original allocation *A*.

### 5.5. EDA for Maximizing NSW and Its Complexity Analysis

In this section, we begin by describing the methodology for determining the optimal Nash welfare value through the application of EDA joint neighborhood search technology. Then, the complexity of the proposed algorithm is analyzed.

**Figure 3:**
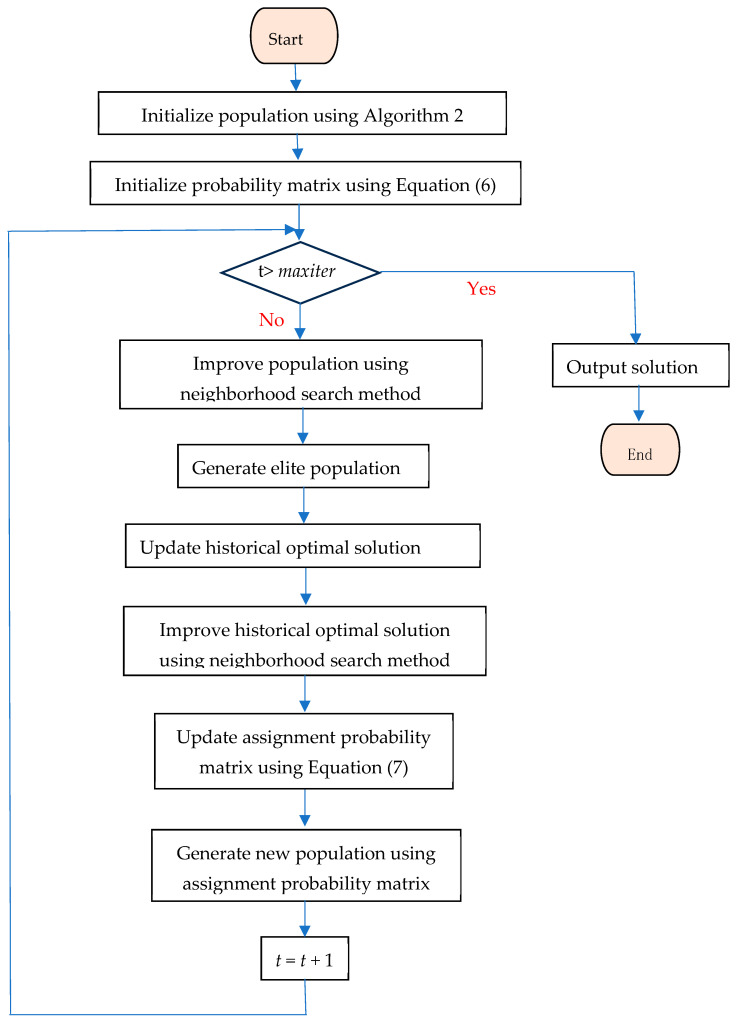
Flow chart for EDA.

The execution flow of maximizing Nash social welfare based on EDAs is shown in Figure 3 and described in Algorithm 3, where *Psize* is the size of the population, *α* is speed learning, *δ* is the percentage of elite solution in the population, and *maxiter* is the maximum iterations of the EDA algorithm. First, the initial population is generated, and the initial assignment probability is established (lines 1–2).

Then, the proposed neighborhood search method is employed to improve each individual (lines 4–6). By evaluating these individuals, those exhibiting a high NSW value are identified and selected as elite candidates. Furthermore, the historical optimal solution is updated in accordance with the elite population (line 7). The neighborhood search method is also utilized to enhance the previously identified optimal solution (line 8). Subsequently, based on the elite population, the assignment probability is updated (line 9).

A new population is generated according to the assignment probability (lines 10–19). There are two ways to allocate goods to an agent. The first way is to allocate goods to an agent according to a roulette based on the assignment probability matrix. The second way is to allocate and assign goods to the agent with the lowest valuation. Each iteration begins by generating a random number *r*, where *r* ∈ (0,1). If *r* exceeds a specified threshold, the first way is employed to allocate goods to the agent (line 14). Conversely, if *r* does not exceed this value, the second way is utilized for the allocation of goods to the agent (line 16).

Finally, when the number of iterations of the algorithm reaches its maximum threshold, the algorithm ceases further iterations and returns the optimal approximation solution (line 21).

The computational complexity of the key operations in the proposed algorithm is outlined as follows: according to Algorithm 2, the complexity associated with generating an initial individual is *O*(*m*). Since the population size of the evolutionary algorithm (EDA) is denoted as *Psize*, the complexity associated with initializing a population is *O*(*Psize* × *m*). The initialization of assignment probability incurs a complexity of *O*(*m* × *n*). Furthermore, sorting the population to select an elite subset has a complexity of *O*(*Psize* × *logPsize*). The process of updating assignment probability exhibits a complexity of *O*(*Psize* × *m* × *n*). Given that calculating the Nash social welfare for an allocation entails a time complexity of *O*(*m* ×*n*), both the random exchanging good strategy and random moving good strategy have complexities represented as *O*(2 × *LNiter* × *m* × *n*) and *O*(2 × *LNiter* × *m* × *n* + *LNiter* × *m*), where *LNiter* is the number of neighborhood searches. Additionally, it can be readily observed that the complexity involved in exchanging goods between the maximum agent and the minimum agent is characterized by *O*(2 × *LNiter* × *m* × *n* + *LNiter* × *n*), while moving goods from maximum agent to minimum agent also maintains a similar complexity profile at *O*(2 × *LNiter* × *m* × *n* + *LNiter* × *n*).
**Algorithm 3:** EDA for Maximizing Nash Social Welfare
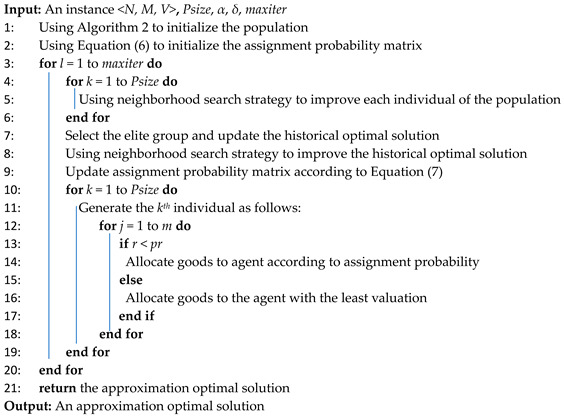


## 6. Simulation Experiment

As far as the author is aware, the previous literature on the Nash social welfare algorithm primarily focused on its design and time complexity, without utilizing datasets to validate the algorithm’s efficiency in solving problems. In this paper, we design two programs to generate datasets of varying scales for Nash social welfare issues. One program addresses a scenario where agents have identical valuations for a single item; the other tackles cases involving different valuations assigned by distinct agents to the same item. To assess the effectiveness of our proposed algorithm in addressing Nash social welfare challenges, we conducted simulation experiments based on these datasets under two specific conditions. The first condition pertains to the NSW problem when all agents share identical valuations. The second condition involves situations where agents possess differing valuations. The proposed algorithm has been implemented using Python 3.2, and all experiments were executed on a PC equipped with an Intel^®^ Core (TM) i7-10510U CPU running at 1.80 GHz (boosting up to 2.30 GHz), along with 16 GB RAM and a Windows 10 operating system.

### 6.1. Simulation Experiment for Agents with Identical Valuation

We have demonstrated that the greedy algorithm proposed in this paper is equivalent to the greedy algorithm presented in [15] when agents possess identical valuations, as discussed in Section 3. In our initial simulation experiment, we examine ten NSW instances where agents share identical valuations. The results of the greedy algorithm (GA) are compared with those obtained from the Estimation of Distribution Algorithm (EDA). The parameters for EDAs are defined as follows: learning rate (*α* = 0.1), the percentage of elite solution in the population (*δ* = 0.1), population size (*Psize* = 50), and maximum iterations (*maxiter* = 20,000). The quantity of agents and goods, as well as the valuation domain for these goods across the NSW instances, is presented in Table 1. For instance, in instance 9, the valuation domain ranges from 1 to 200, with a total of 50 agents and 500 goods involved. To illustrate the universal effectiveness of our algorithm, we randomly generate a value within the valuation domain for a good. The values assigned to a good follow a uniform distribution.

The approximation optimization results for both GAs and EDAs are presented in Table 2; specifically, the GA column displays outcomes derived from GAs while the EDA column reflects those obtained through EDAs. The simulation results indicate that when agents have identical valuations, the approximate optimization achieved by EDAs outperforms that attained by GAs. *NSW*(*A^GA^*) and *NSW*(*A^EDA^*) denote the approximate optimization of EDAs and Gas, respectively, while *NSW*(A^*^) represents the actual maximum Nash social welfare. It has been demonstrated that $\frac{\mathrm{NSW}(A^{\mathrm{GA}})}{\mathrm{NSW}(A^{*})}\approx \frac{1}{1.0607}$ as shown in [15]. Additionally, given that *NSW*(*A^EDA^*) > *NSW*(*A^GA^*), this leads to the conclusion that

(19) $$\mathrm{NSW}(A^{\mathrm{EDA}})>\frac{1}{1.0607}\mathrm{NSW}(A^{*})$$

### 6.2. Simulation Experiment for Agents with Different Valuations

In this section, we examine ten instances of agents with different valuations and compare the results of the greedy algorithm with those of the distribution estimation algorithm. The parameters for the EDA are specified as follows: learning rate (*α* = 0.1), the percentage of elite solution in the population (*δ* = 0.1), population size (*Psize* = 60), and maximum iterations (*maxiter* = 3000). The number of agents and goods, as well as the domain of good valuation for the NSW instances, are presented in Table 3.

The EDA algorithm was executed ten times for each instance, with the results displayed in Table 4.

In Table 5, the GA column presents the outcomes from the Genetic Algorithm (GA), while the ED (av) column indicates the average NSW obtained from the EDA. Additionally, the columns labeled EDA (min), EDA (max), and EDA (sd) represent, respectively, the maximum NSW achieved by the EDA; the minimum NSW attained by the EDA; and finally, the standard deviations for the EDA. The results indicate that the Nash social welfare value computed by the Estimation of Distribution Algorithm (EDA) surpasses that obtained through the Genetic Algorithm (GA). The differences between the average Nash social welfare (NSW) values of the EDA and those from the GA are 38.64, 8.57, 38.28, 2.84, 3.89, 20.15, 3.49, 28.71, 46.53, and 11.22, respectively. This clearly demonstrates that the EDA outperforms the greedy algorithm. Furthermore, the standard deviations for the EDA across all instances are recorded as follows: 1.57, 0.22, 4.60, 1.53, 1.19, 4.91, 0.20, 4.61, 2.46, and 1.82, respectively. This suggests that the results produced by EDAs exhibit minimal variation across different runs. Consequently, the proposed EDA demonstrates commendable stability in addressing the problem of Nash social welfare.

### 6.3. Sensitivity Analysis of Algorithm Parameters

The EDA algorithm is characterized by three essential parameters: the population size (*Psize*), the learning rate (*α*), and the proportion of elite groups (*δ*). In this study, we utilize the tenth instance presented in Section 6.2 to investigate how variations in these three parameter settings influence the solution outcomes of the algorithm through an experimental design approach.

Each parameter has three levels, with the values for each level presented in Table 6. Based on the number of parameters and their respective levels, we selected an orthogonal experiment design with a scale of 9. For each combination of parameters, the algorithm was executed independently ten times. The various combinations along with their average NSW values are displayed in Table 7.

**Figure 4:**
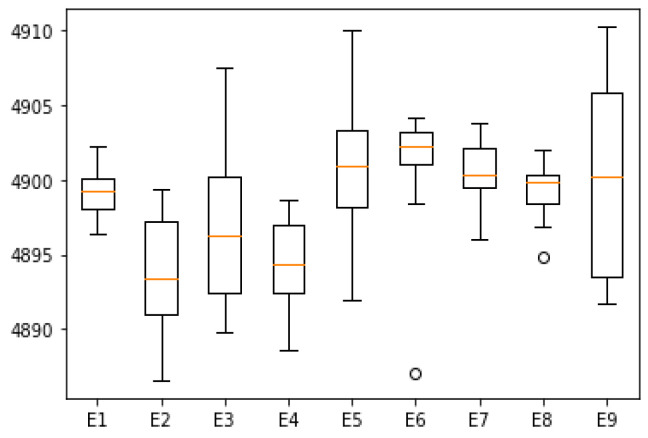
Boxplots for each parameter combination.

In the case of parameter combination 5 (*Psize* = 80, *α* = 0.4, *δ* = 0.1), the algorithm obtains the largest average value of NSW. Conversely, for parameter combination 2 (*Psize* = 80; *α* = 0.2; *δ* = 0.15), the average value of NSW is at its lowest. The NSW values corresponding to each parameter combination are presented in Table 8. Additionally, Figure 4 illustrates the boxplots of NSW for all parameter combinations examined.

It can be seen from Table 8 that the maximum value of NSW achieved by the algorithm for parameter combinations 3, 5, and 9 surpasses that of the other combinations. For combination 6, the median is the highest, while combination 2 exhibits the lowest median. The range and significance of each parameter are presented in Table 9.

It is evident that the learning rate has the most substantial impact on algorithm performance. An increase in the learning rate may lead to premature convergence; thus, the value of NSW does not necessarily rise with an increasing *α* value. Therefore, selecting an appropriate learning rate is crucial. The second most significant parameter is the proportion of elite groups. Experimental results indicate that as *δ* increases, there tends to be a decrease in NSW values. Conversely, population size appears to have minimal influence on algorithm performance. Nonetheless, experimental data still suggest that larger populations yield higher NSW outcomes.

## 7. Conclusions and Future Works

In this paper, we present two types of algorithms designed to address the Nash social welfare problem. We introduce a more general greedy algorithm that is applicable to both scenarios where agents have identical valuations and those with differing valuations. Furthermore, we demonstrate that the greedy algorithm is a specific instance of our proposed approach. Distinct from existing methods, we investigate the use of distribution estimation algorithms (EDAs) as a means to tackle the Nash social welfare issue. To validate our findings, we conducted experiments comparing the EDA with the greedy algorithm, revealing that the EDA consistently outperforms its counterpart. In the first set of experiments, both the EDA and the greedy algorithm were executed on ten instances involving agents with identical valuations. The experimental results indicate that the EDA achieves solutions at least 0.943 times closer to the actual optimal solution compared to those obtained by the greedy algorithm. In the second set of experiments, we consider cases where agents possess different valuations; comparison results illustrate that the EDA yields higher Nash social welfare outcomes than those produced by the greedy algorithm.

However, the EDA predicts the optimal region by employing sampling and statistical learning techniques within the search space. This approach necessitates substantial computational resources, particularly when dealing with a large number of samples, resulting in elevated calculation costs. Moreover, even if the effectiveness of the EDA in providing solutions is commendable, we cannot guarantee the optimality of EDAs in polynomial time, which still needs further research. Future work will primarily focus on the following three aspects: First, various intelligent algorithms, such as the Ant Colony Optimization algorithm (ACO), Whale Optimization Algorithm (WOA), Quantum-Inspired Evolutionary Algorithm (QIEA), and Spider Monkey Optimization algorithm (SMO), will be employed to address the Nash social welfare problem. Second, the Nash social welfare problem will be reformulated as a multi-objective optimization challenge, utilizing multi-objective optimization algorithms to maximize Nash social welfare. Finally, we will explore how to apply intelligent algorithms to compute effective approximations of the Nash social welfare problem using truthful yet non-wasteful mechanisms.

## Figures and Tables

**Table 1:** Ten instances with identical valuation.

No.	*n*	*m*	Valuation Domain
1	10	30	[1, 20]
2	10	30	[1, 500]
3	10	100	[1, 20]
4	20	200	[1, 100]
5	30	200	[1, 200]
6	30	300	[1, 500]
7	40	400	[1, 100]
8	40	500	[1, 500]
9	50	500	[1, 200]
10	80	600	[1, 500]

**Table 2:** Simulation results of identical valuation.

No.	GA	EDA
1	27.244	27.247
2	841.057	842.244
3	102.831	102.832
4	496.494	496.497
5	700.438	700.442
6	2509.859	2059.873
7	520.517	520.522
8	3174.779	3174.784
9	985.616	985.617
10	1927.055	1927.058

**Table 3:** Ten instances with different valuations.

No.	*n*	*m*	Valuation Domain
1	30	300	[10, 500]
2	20	300	[1, 100]
3	40	400	[100, 1000]
4	50	400	[100, 500]
5	50	500	[10, 200]
6	60	300	[1, 1000]
7	60	400	[1, 100]
8	40	400	[1, 1000]
9	70	300	[1, 1000]
10	80	400	[1, 1000]

**Table 4:** A comparative table of results for GAs and EDAs.

No.	GA	EDA (av)	EDA (max)	EDA (min)	EDA (sd)
1	4782.99	4821.63	48,824.88	4819.76	1.57
2	1410.84	1419.41	1419.70	1418.82	0.22
3	9642.84	9681.12	9689.73	9673.22	4.60
4	3913.77	3916.61	3919.86	3914.47	1.53
5	1948.12	1952.01	1954.49	1950.23	1.19
6	4857.06	4877.21	4886.27	4867.82	4.91
7	647.71	651.20	651.38	650.68	0.20
8	9696.25	9724.96	9733.39	9719.51	4.61
9	4142.68	4189.21	4192.44	4184.66	2.46
10	4888.08	4899.30	4902.27	4896.33	1.82

**Table 5:** The results of EDAs.

No.	Run1	Run2	Run3	Run4	Run5	Run6	Run7	Run8	Run9	Run10
1	4819.76	4820.49	4820.05	4822.79	4820.49	4824.88	4822.83	4820.39	4822.07	4822.59
2	1419.70	1418.82	1419.37	1419.44	1419.38	1419.61	1419.39	1419.38	1419.57	1419.44
3	9683.66	9680.63	9673.22	9680.11	9682.80	9676.03	9678.95	9689.73	9686.66	9679.43
4	3916.60	3917.78	3919.86	3914.72	3915.79	3917.66	3917.40	3915.99	3915.80	3914.47
5	1952.42	1951.30	1953.70	1951.34	1954.49	1951.32	1951.49	1950.23	1952.21	1951.62
6	4879.84	4877.96	4886.27	4875.53	4881.06	4867.82	4874.70	4871.95	4876.49	4880.52
7	651.34	651.32	650.68	651.05	651.15	651.30	651.18	651.27	651.38	651.34
8	9719.51	9733.39	9731.66	9725.95	9722.99	9722.32	9720.66	9724.17	9728.69	9720.27
9	4192.04	4191.17	4188.88	4184.66	4189.38	4190.57	4186.52	4192.44	4186.42	4189.99
10	4896.33	4897.77	4899.31	4897.50	,902.27	4898.71	4900.38	4899.41	4902.16	4899.16

**Table 6:** The level values of algorithm parameters.

Parameters	Level		
	1	2	3
Psize	60	80	100
α	0.1	0.2	0.4
δ	0.1	0.15	0.2

**Table 7:** Orthogonal table and average NSW.

No.	Level			The Average NSW
	*Psize*	*α*	*δ*	
1	1	1	1	4899.30
2	2	2	2	4893.82
3	3	3	3	4897.14
4	1	2	3	4894.35
5	2	3	1	4901.24
6	3	1	2	4900.61
7	1	3	2	4900.50
8	2	1	3	4899.24
9	3	2	1	4900.32

**Table 8:** Experimental results of different parameter combinations.

No.	Run1	Run2	Run3	Run4	Run5	Run6	Run7	Run8	Run9	Run10
1	4896.33	4897.77	4899.31	4897.50	4902.27	4898.71	4900.38	4899.41	4902.16	4899.16
2	4896.43	4893.70	4890.58	4892.10	4893.16	4898.66	4899.35	4886.56	4890.17	4897.50
3	4907.59	4904.40	4895.75	4891.47	4889.76	4890.77	4900.57	4896.67	4899.40	4895.05
4	4888.62	4892.12	4898.69	4897.43	4895.42	4895.19	4898.07	4893.25	4891.24	4893.44
5	4896.91	4910.06	4903.40	4899.47	4897.71	4907.68	4901.49	4903.23	4900.49	4891.97
6	4903.37	4902.54	4901.00	4903.83	4902.69	4901.92	4887.02	4901.10	4904.23	4898.39
7	4901.79	4903.87	4899.45	4899.76	4902.24	4900.25	4903.55	4897.62	4896.06	4900.37
8	4900.00	4900.25	4899.74	4900.71	4894.83	4898.06	4896.81	4899.65	4902.01	4900.33
9	4905.75	4903.39	4894.64	4893.17	4910.26	4905.84	4896.96	4908.34	4891.68	4893.14

**Table 9:** The average values of NSW for the parameters.

	*Psize*	*α*	*δ*
1	4898.05	4899.72	4900.29
2	4898.10	4896.16	4898.31
3	4899.34	4899.63	4896.91
Range	1.29	3.56	3.38
Grade	3	1	2

## Data Availability

The data that support the findings of this study are available from the corresponding author upon request. There are no restrictions on data availability.
